# Recent developments on potential new applications of emetine as anti-cancer agent

**DOI:** 10.17179/excli2016-280

**Published:** 2016-05-10

**Authors:** Philip F. Uzor

**Affiliations:** 1 Department of Pharmaceutical and Medicinal Chemistry, University of Nigeria, Nsukka, 410001, Nigeria

## ⁯

Dear Editor,

Cancer remains one of the leading causes of global morbidity and mortality, with approximately 14 million new cases and 8.2 million cancer related deaths in 2012 (Stewart and Wild, 2014[37]). Treatment protocols include radiation, surgery, chemotherapy, hormone therapy, immunotherapy and targeted therapy (American Cancer Society, 2015[6]). While chemotherapy is one of the key strategies against cancer, the available drugs are frequently fraught with toxicity and increased frequency of tumor relapse (Gaziano et al., 2016[12]). This calls for an urgent need for more effective anti-tumor agents especially from phytochemicals which are known to be of lower toxicity and cost (Reddy et al., 2003[35]). A wide variety of phytochemicals, particularly alkaloids, have been investigated in recent times in the quest for more effective and safer antitumor agents (Lu et al., 2012[26]; Kharwar et al., 2011[20]). Interestingly, several important anti-tumor alkaloidal drugs have been isolated from medicinal plants including the vinca alkaloids, vinblastine and vincristine, isolated from the Madagascar periwinkle, *Catharanthus roseus* (Noble et al., 1958[31]; Johnson et al., 1959[17]; Svoboda, 1961[40]) as well as paclitaxel, isolated from *Taxus brevifolia *(Wani et al., 1971[44]). One effective strategy employed by scientists in this regard is the investigation of known drugs for novel biological effects, the so called 'drug repositioning'. One of such known drugs that have been shown to possess anti-tumor activity is the alkaloidal amoebicidal drug, emetine (EMT). 

**Figure 1:**
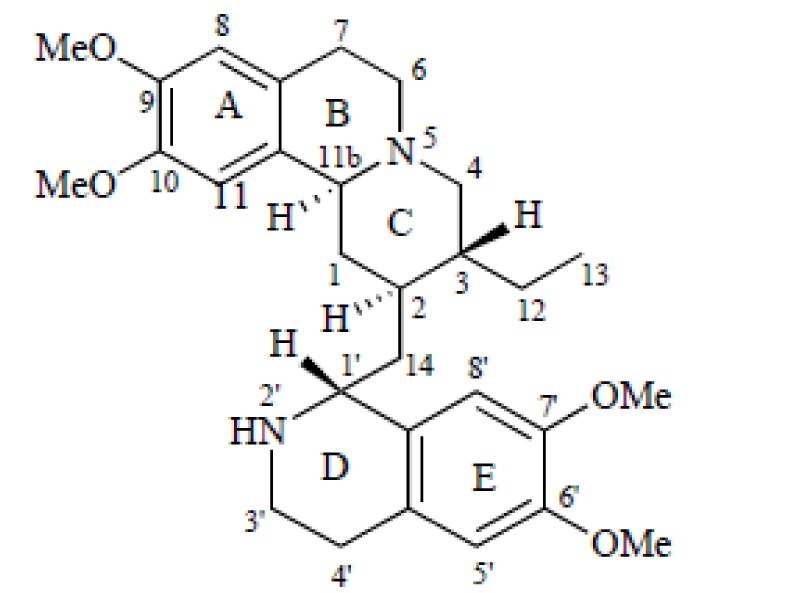
Chemical structure of EMT

EMT, chemically designated as 2S,3R,11bS)-2-{[(1R)-6,7-dimethoxy-1,2,3,4-tetrahydroisoquinolin-1-yl]methyl}-3-ethyl-9,10-dimethoxy-2,3,4,6,7,11b-hexahydro-1H-pyrido [2,1-a]isoquinoline (Figure 1(Fig. 1)), is an isoquinoline alkaloid which occurs in the families of Alangiaceae, Icacinaceae, and Rubiaceae. The major source of EMT and its analogs is *Psychotria ipecacuanha* Stokes (Rubiaceae) which is also known as *Cephaelis ipecacuanha *A. Rich (ipecac) where it is the principal alkaloid (Wiegrebe et al., 1984[45]). It is clinically used (as a dihydrochloride) in the treatment of amoebiasis, a protozoan infection (Vedder, 1912[42]) and it has emetic properties. It is reportedly a protein synthesis inhibitor in eukaryotes (Grollman, 1968[13]). The biosynthesis of EMT and cephaeline (another alkaloid found in ipecac) comes from two main biosynthesis pathways, the biosynthesis of dopamine from L-tyrosine and that of secologanin from geranyl diphosphate (Cheong et al., 2011[9]; Nomura et al., 2010[32]).

The anti-cancer effect of EMT was first reported on malignant human tumors in 1918 by Lewisohn (1918[25]) but since he was unable to reproduce this effect in laboratory animals, he concluded that the drug had no anti-tumor properties and that the tumor regression must have been spontaneous. However, in the following year, Van Hoosen (1919[41]) further reported the remission of various malignancies in a number of patients by EMT. This is followed in later years by reports of effectiveness of EMT in rat Yoshida sarcoma (Isaka, 1950[16]), intra-abdominal and retroperitoneal nonspecific granulomas (Grollman, 1965[13]) and in murine leukemia (Jondorf et al., 1970[18]). Besides, the potency of an analogue of EMT, dehydroemetine, was also shown in chronic granulocytic leukemia (Abd-Rabbo, 1966[2]), various malignancies (Abd-Rabbo, 1969[1]) as well as in Hodgkin's disease and rectal adenocarcinoma (Wyburn-Mason, 1966[46]). Based on these reports, phase I and II clinical trials with EMT were done in the early 1970s (Panettiere and Coltman, 1971[34]; Street, 1972[38]; Mastrangelo et al., 1973[27]; Siddiqui et al., 1973[36]; Moertel et al., 1974[29]; Kane et al., 1975[19]). The drug was, however, discontinued from the clinical trials (Von Hoff et al., 1977[43]) due to its very narrow therapeutic index, cardiac toxicity and other adverse effects which were also observed in the treatment of amoebic patients (Knight, 1980[22]). Since then the drug has been used in *in vitro* experimental studies requiring inhibition of protein biosynthesis (Akinboye et al., 2012[5]). The data from these recent studies have further shown EMT as a modulator of different cancer related biological pathways. In fact, excellent review by Akinboye and Bakare (2011[3]) has shown that EMT exhibits its anti-tumor effect by apoptosis through such mechanisms as inhibition of protein biosynthesis, DNA interaction and regulation of pro-apoptotic factors. In more recent years also, various studies have further investigated the role of EMT in cancer growth arrest and its biological targets using a variety of human carcinoma cell lines. New derivatives have also been synthesized and reported to be efficacious but less toxic to normal cells. Also the drug has been investigated in combination with other agents to assess their anti-tumor synergistic effect which will warrant reduction in its dose. These studies are geared towards bringing back EMT or its derivatives to the clinical limelight in cancer chemotherapy. The present report summarizes these more recent anti-tumor updates on EMT (Table 1(Tab. 1); References in Table 1: Sun et al., 2015[39]; Kim et al., 2015[21]; Mayank and Jaitak, 2016[28]; Han et al., 2014[15]; Myhren et al., 2014[30]; Foreman et al., 2013[10]; Akinboye et al., 2012[5]; Larrson et al., 2012[24]; Pan et al., 2011[33]; Kong et al., 2010[23]). It is hoped that this report will further spur research interests on EMT and its structural modifications towards potential application in cancer chemotherapy.

## Figures and Tables

**Table 1:**
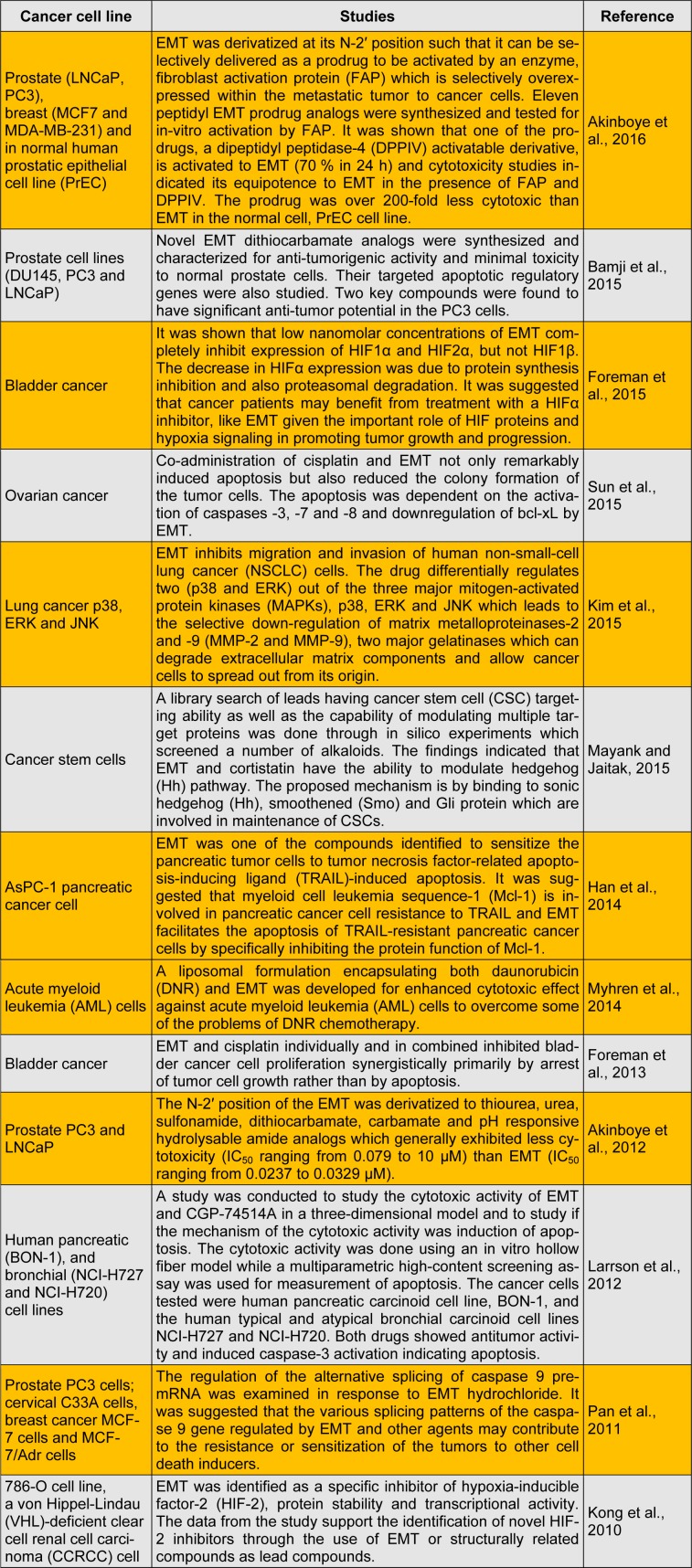
Recent studies on EMT in relation to anti-cancer effect
